# Impact of Resident Doctors’ Strike on Psychological Outcomes Among Paramedics in Teaching-Hospital Emergency Departments: A Nationwide Multicenter Survey

**DOI:** 10.3390/healthcare14040480

**Published:** 2026-02-13

**Authors:** Keun-Young Kim, Yong-Seok Kim, Seong-Ju Kim, Geon-Uk Ryu, Hyeong-Tae Kim, Chan-Young Kang, Yun-Deok Jang

**Affiliations:** 1 Department of Paramedicine, Daegu Health College, Daegu 41453, Republic of Korea; silverlove93@naver.com; 2 Department of Paramedicine, Konyang University, Daejeon 35365, Republic of Korea; ys031113@gmail.com; 3 Department of Paramedicine, Tongmyong University, Busan 48520, Republic of Korea; superemt@naver.com; 4 Korean Association of Emergency Medical Technician, Ohsong 28159, Republic of Korea; gw4642@naver.com; 5 Department of Emergency Medicine, Wonju Severance Christian Hospital, Wonju 26426, Republic of Korea; kkkhht@hanmail.net (H.-T.K.); chlrhkcy@naver.com (C.-Y.K.)

**Keywords:** paramedics, strike, job stress, self-efficacy, job satisfaction

## Abstract

**Background/Objectives:** In March 2024, a resident doctor’s strike in South Korea created staffing gaps in teaching-hospital emergency departments. The purpose of this study was to evaluate pre- and post-strike changes in job stress, self-efficacy, job satisfaction, and job performance confidence among paramedics who work in hospitals and to compare patterns of change by career stage. **Methods:** Paramedics who work in hospitals designated as regional and local emergency medical centers completed a structured 41-item questionnaire across four domains on a 5-point Likert scale. Retrospective pre- and post-strike ratings were analyzed using paired *t*-tests. Subgroup analyses compared senior (≥5 years) and junior (1–2 years) paramedics. **Results:** Job stress increased after the strike, including additional task instruction (2.8 ± 0.9 to 3.6 ± 1.0), insufficient rest (3.1 ± 0.7 to 3.9 ± 0.9), and multitasking burden (3.3 ± 0.8 to 4.1 ± 0.9). Self-efficacy declined modestly (confronting difficult tasks: 3.9 ± 0.9 to 3.6 ± 0.9). Job satisfaction decreased (reward after work: 3.9 ± 0.7 to 3.5 ± 0.9), while turnover intention increased (2.7 ± 1.0 to 3.9 ± 0.9). Performance confidence showed minimal change (competence: 4.4 ± 0.6 to 4.3 ± 0.8). Subgroup findings were similar in seniors and juniors, with stress increasing and self-efficacy decreasing overall. **Conclusions:** Resident workforce shortages increased job stress among paramedics working in teaching-hospital emergency departments and heightened negative perceptions of their work. To prepare for similar workforce crises in the future, it is necessary to revise and realign the legal scope of practice to reflect paramedics’ roles and responsibilities in real-world settings while simultaneously establishing the policy and institutional infrastructure needed to support these changes.

## 1. Introduction

Korean paramedics are specialized professionals responsible for providing consultation, rescue, transport, and emergency care at the scene where an emergency occurs, and they play a frontline role in saving lives in both social disasters and everyday accidents [1,2]. Paramedics complete formal professional education at the university level and are licensed through a national certification examination [2]. Under physician supervision, they are qualified to perform advanced emergency procedures such as advanced cardiac life support, advanced airway management, establishment of intravenous access, and trauma care within the legally defined scope of practice [2,3,4].

A substantial proportion of paramedics in South Korea also work in hospital-based emergency departments and serve as key personnel in the care of emergency patients [3,5].

In the emergency department, paramedics contribute across diverse domains, including triage support and initial assessment assistance, emergency interventions under supervision, patient transport and monitoring, and clinical assistance through collaboration with emergency physicians, as well as selected administrative and coordination duties [4,5,6]. When inter-hospital transfer is required, they accompany patients in ambulances, closely monitor patient conditions, and implement physicians’ orders during transport [1,3]. Korean paramedics therefore constitute an essential component of the national emergency medical system and contribute to public safety and the protection of life [1,2,3]. Notably, the division of labor between paramedics and resident physicians in Korean teaching-hospital emergency departments reflects a distinct historical and institutional context [5,6]. Following the establishment and expansion of emergency medicine residency training and university-based paramedic education, paramedics’ in-hospital roles evolved in many emergency medical centers and increasingly involved advanced clinical assistance under physician supervision, while remaining within legally defined limits [2,3,4,5,6].

Consequently, some operational tasks may overlap with activities commonly supported by residents, whereas diagnostic and therapeutic decision-making authority and ultimate clinical responsibility remain physician-led [4,5,6]. Emergency departments are characterized by high levels of unpredictability and urgency and are widely recognized as workplaces with substantial workload and psychological pressure even under routine conditions [7]. In this environment, the psychological status of frontline personnel may influence not only individual health and job sustainability but also patient safety and the quality of care [7,8].

In particular, psychological factors experienced during job performance, including job stress, self-efficacy, job satisfaction, and confidence in job performance, may affect coping in crisis situations and team-based task performance [9]. In this context, the collective action by resident doctors that began in February 2024 created substantial staffing gaps in many teaching hospitals in South Korea and triggered rapid reorganization of clinical assistance and operational processes [8,9].

Contemporaneous reports described large-scale walkouts and resignations among trainee doctors and warned of delays in patient treatment during the disruption [7,8].

As resident staffing was reduced, hospitals relied more heavily on other emergency department personnel to maintain operational continuity, potentially increasing perceived responsibility and psychological burden among paramedics working in hospital-based emergency departments [4,6,9]. Job stress can be defined as harmful physical and emotional responses that occur when job demands do not match the capabilities, resources, or needs of the worker [10].

Heavy workload, shift work, and interprofessional conflict have been identified as major stressors among emergency care workers [11,12]. High job stress is associated with burnout, increased turnover intention, decreased job satisfaction, and adverse implications for patient safety and quality of care, underscoring the need for organizational and system-level management [13]. In contrast, self-efficacy, defined as an individual’s belief in their capability to successfully perform specific tasks, is a psychological resource that supports coping and sustained performance in high-risk environments [11].

Confidence in clinical job performance has also been reported as a key factor influencing routine job performance, engagement, and job sustainability [12]. Prior research among hospital-based emergency workforce and related clinical settings suggests that higher stress is linked to poorer work outcomes, while self-efficacy and related psychological resources are linked to better job satisfaction and retention [11,12,13]. Importantly, the same external disruption may not affect all paramedics equally [2,14]. Because clinical experience, coping resources, and role responsibilities differ by career stage, senior and junior paramedics may exhibit different psychological responses in the same event [2,14]. Therefore, comparing psychological responses by career stage can help identify groups more strongly affected and inform targeted support strategies [2,14]. Despite this, evidence remains limited regarding comprehensive assessment of job stress, self-efficacy, job satisfaction, and confidence in job performance among paramedics working in teaching-hospital emergency departments in the context of large-scale resident workforce disruptions [12,14]. Quantitatively characterizing psychosocial changes among frontline personnel during rapid role redistribution has academic and policy significance for workforce planning and support within emergency care systems [12,13,14].

Accordingly, this study aims to examine changes in key psychological factors, including job stress, self-efficacy, job satisfaction, and confidence in job performance, among paramedics working in teaching-hospital emergency departments who experienced the resident doctors’ collective action [7,8,9].

Additionally, we assess differences in psychological responses between senior and junior paramedics [2,14].

Through these analyses, we seek to provide evidence on how role and workflow reorganization influences paramedics’ psychological status and to offer foundational data for future psychological support measures and workforce policy development [12,13,14].

## 2. Materials and Methods

### 2.1. Study Design

This study is a prospective, questionnaire-based observational study conducted to quantitatively analyze the impact of the resident doctor’s strike that began in March 2024 on the psychological status of paramedics working in emergency departments of teaching hospitals. The primary aim of this study was to empirically identify changes in job stress and self-efficacy among paramedics in the absence of resident physicians and to provide foundational data for developing strategies for emergency medical workforce management.

The study is described as prospective because the research protocol was predefined and Institutional Review Board (IRB) approval was obtained prior to participant recruitment and data collection. After IRB approval, eligible paramedics were enrolled and surveyed forward in time within the predefined study period. In addition to assessing current psychological status at the time of survey administration, pre-strike status was assessed using a standardized “before-strike” reference period (predefined in the questionnaire) based on participants’ self-reports, enabling within-participant comparison between before-strike and post-strike periods.

This study was conducted after obtaining approval from the Institutional Review Board (IRB). To collect data, a survey was administered to paramedics working at regional emergency medical centers and local emergency medical centers registered under the “Hospital Paramedics Association,” affiliated with the Korean Association of Paramedics (IRB No. 2024-04-0011).

### 2.2. Study Population

This study was conducted among paramedics working at regional emergency medical centers and local emergency medical centers across the Republic of Korea. The target population was defined using the Ministry of Health and Welfare’s Emergency Medical Resources Survey and the most recent workforce data provided by the Korean Association of Paramedics. As of 31 December 2024, Korea had 26,992 licensed EMT paramedics, and 4143 were employed in medical institutions. This employment category includes emergency departments of designated regional and local emergency medical centers and therefore represents the principal workforce pool relevant to this study. Because national workforce statistics are typically reported by broad employment sectors, the number of EMT paramedics working specifically in emergency departments of emergency medical centers is not always available as a single national aggregate. We therefore used the medical institution workforce figure to contextualize the sampling frame for ED-based paramedics.

A stratified sampling frame was constructed based on regional distribution and center type, and the sample size was allocated proportionally to the size of each stratum. Sample size was determined primarily by the stratified design and feasibility considerations rather than a single-effect power calculation. To ensure broad institutional coverage, we recruited participants from 100 institutions and selected two paramedics from each participating institution. This yielded an initial target sample of 200. After providing an explanation of the study purpose and procedures, 161 individuals voluntarily consented to participate and were included in the final analysis, corresponding to a response rate of 80.5%.

Eligible participants were licensed EMT paramedics who were currently assigned to and working in the emergency department of a participating emergency medical center. Participants were required to have worked in the emergency department for at least six months to ensure adequate exposure to routine workflows before the strike and to operational changes after the strike. They also needed to be able to complete the questionnaire in Korean and to provide informed consent.

Career stage was operationalized using years of professional experience as an EMT paramedic. Junior paramedics were defined as those with fewer than five years of experience, and senior paramedics were defined as those with five years of experience or more. This threshold was selected to distinguish early-career from more established clinical practice in emergency department operations and to support subgroup comparisons.

Exclusion criteria included not currently working in an emergency department, such as ward-only, outpatient-only, or administrative-only roles. Unlicensed personnel such as students or trainees and non-paramedic healthcare professionals were excluded. We also excluded individuals with less than six months of employment in the emergency department or with prolonged absence during the study period that would preclude reliable comparison. Incomplete or invalid questionnaires were excluded, including missing responses for key outcome variables, as predefined in the analysis plan.

### 2.3. Study Protocol

Data collection was conducted using an online survey. With the cooperation of the ‘Korean Association of Paramedics’, the survey link was distributed to eligible participants, and the survey period lasted 17 days, from 17 April to 2 May 2024. Participants were paramedics working at emergency departments of teaching hospitals. Before responding, they were fully informed of the study purpose and the principles of voluntary participation, anonymity, and confidentiality, and they provided consent to participate.

The questionnaire was developed using ‘Google Forms’ and was designed to be accessible on both mobile devices and personal computers. It consisted of a total of 41 items: 11 items on job stress after the resident doctors’ collective action, 14 items on self-efficacy after the collective action, 10 items on job satisfaction after the collective action, and 6 items on confidence in job performance after the collective action. Responses were primarily measured on a 5-point Likert scale (1 = strongly disagree to 5 = strongly agree). Prior to analysis, responses were screened to exclude careless or duplicate submissions. The final dataset was used as foundational evidence to assess psychological changes among paramedics during the period of resident physician shortages.

Because this study assessed paramedics’ psychological changes like job stress, self-efficacy, and confidence in job performance retrospectively based on a specific event period, procedures were implemented to minimize potential recall bias. First, to anchor the reference period clearly, participants were provided with the specific timeframe (year and month) of the resident doctors’ strike and concrete examples of emergency department operational conditions during that period to facilitate recall of the same event. Second, job stress, self-efficacy, and confidence in job performance were measured using standardized instruments with established reliability and validity in prior studies, thereby reducing subjective reinterpretation. Third, the recall window was limited to within four weeks, and participants were instructed to respond within this short recall period to minimize memory distortion. Fourth, before starting the survey, participants received cognitive instructions to respond based on factual experiences; for items asking about specific behaviors or situations, behavioral criteria were provided to prevent reliance on vague memories.

Finally, anonymity and confidentiality were emphasized to reduce distortion due to social desirability and to encourage honest self-reporting. These procedures were intended to systematically reduce information bias inherent in retrospective self-reports and to strengthen the internal validity of the study findings.

### 2.4. Study Materials

#### 2.4.1. General Characteristics Survey

In this study, eight items were developed to capture participants’ sociodemographic characteristics and basic job-related information. These items assessed sex, age, marital status, educational level, total years of work experience, type of affiliated institution, employment status, and the number of paramedics employed at the institution. These variables were used as baseline characteristics to examine differences in perceived job-related changes across respondent subgroups.

Content validity was established through expert review. An expert panel with extensive experience in emergency medical services surveys evaluated the relevance and clarity of each item. The panel consisted of one board-certified emergency physician and three professors specializing in paramedic science. Each expert rated item relevance using a four-point scale. Item-level content validity indices were calculated as the proportion of experts rating an item as relevant. Scale-level content validity was then computed as the average of item-level indices. The mean content validity index was 0.92, indicating high content validity.

Reliability was assessed using internal consistency. Cronbach’s alpha for the eight baseline items was 0.81, suggesting acceptable internal consistency for the scale.

#### 2.4.2. Material for Measuring Changes in Job Stress Before and After Resident Doctors’ Strike

Job stress was measured using an 11 item self-report scale designed to reflect changes in the work environment experienced by paramedics following the resident doctors’ strike. The scale was adapted from existing job stress instruments used in clinical settings and was revised to incorporate stressors specific to emergency department practice. To establish content validity, an expert panel consisting of one board-certified emergency physician, two professors of emergency medical technology, and two paramedics with more than ten years of clinical experience reviewed each item for relevance and contextual appropriateness. Experts rated item relevance using a four-point scale, and the item-level content validity index was calculated as the proportion of experts rating an item as relevant.

During this process, the items were reconstructed to focus on stressors that were expected to substantially increase in the absence of residents. These included a surge in workload, time pressure, insufficient rest, concurrent task directives, and role shifting. Representative items included “I am often assigned additional tasks before I can finish my current work,” “My workload has increased markedly,” and “I am not provided with sufficient rest during work,” which directly captured the stressors paramedics reported experiencing during that period.

After item development, content validity was quantified, and all items met the predefined retention criterion with an item-level content validity index of 0.80. A pilot test with 20 participants was conducted to evaluate item clarity, response variability, and completion time, and feedback from the pilot test was incorporated into the final questionnaire. In the present study, internal consistency reliability was high, with a Cronbach’s alpha of 0.89. We considered general stress instruments such as the Perceived Stress Scale. However, because our objective was to capture strike-related and ED-specific job stressors linked to task redistribution, we used an adapted job stress measure tailored to the emergency department context. Total scores ranged from 1 to 5, with 3 indicating a mid-level response.

#### 2.4.3. Self-Efficacy Measurement After Resident Doctors’ Strike

Self-efficacy was assessed to evaluate paramedics’ perceived confidence in their ability to perform required tasks and cope effectively in emergency department practice under conditions of resident workforce shortages. Self-efficacy was measured using a 14-item self-report scale adapted from previously validated self-efficacy instruments used in healthcare professionals, with minor wording adjustments to fit the emergency medical center context and the strike-related work environment. Participants rated each item on a 5-point Likert scale, where higher scores indicated greater self-efficacy. Total scores ranged from 1 to 5, with 3 indicating a mid-level response.

The scale items were designed to capture key aspects of self-efficacy relevant to ED practice, including perceived ability to complete assigned tasks, confidence in executing plans, persistence when facing difficult tasks, and reduced anxiety about one’s abilities during duty performance. For transparency and interpretability, we present a subset of representative items that reflect these domains in the main text.

Content validity was evaluated through expert review for relevance and clarity. The mean content validity index for the self-efficacy scale was 0.90, indicating high content validity. Reliability was assessed as internal consistency reliability, and Cronbach’s alpha was 0.79, suggesting acceptable internal consistency in the study sample.

#### 2.4.4. Changes in Job Satisfaction After Resident Doctors’ Strike

Job satisfaction was assessed to evaluate paramedics’ overall satisfaction with their current job. We used a 10-item job satisfaction scale based on an instrument validated in prior research. Several items were revised and additional items were included to reflect the work environment of emergency medical centers and organizational changes following the resident doctors’ strike. Content validity was evaluated through expert review. An expert panel consisting of one board-certified emergency physician, two professors of paramedicine, and two paramedics with more than ten years of clinical experience assessed each item for appropriateness, clarity, and relevance to real-world practice.

The items covered major domains of job satisfaction, including perceived accomplishment from work, positive perceptions of the organization and workplace, commitment to remain in the job, and intention to leave. Representative items included “I feel a sense of accomplishment after finishing a day’s work,” “I am satisfied with my current workplace,” and “If better conditions were available, I would be willing to change jobs.” The scale was designed to capture affective satisfaction as well as organizational attitudes and behavioral intentions.

After item development, the content validity index was calculated. All items met the predefined retention criterion and demonstrated an item-level content validity index of 0.88. A pilot test was then conducted with 15 individuals with characteristics similar to those of the study participants to evaluate item comprehensibility, response consistency, and completion time. Based on the pilot results, minor wording revisions were made. In the present study sample, internal consistency reliability of the job satisfaction scale was acceptable, with a Cronbach’s alpha of 0.867. Total scores ranged from 1 to 5, with 3 indicating a mid-level response.

#### 2.4.5. Changes in Performance Confidence After Resident Doctors’ Strike

Performance confidence was assessed to evaluate the extent to which paramedics felt confident in their ability to perform in actual clinical settings. We used a six-item performance confidence scale adapted from a previously published instrument used among clinical professionals. Several items were revised and additional items were included to fit the emergency medical center context and to reflect strike-related conditions that may affect confidence. These conditions included increased workload, more frequent procedures, and greater demands for independent clinical judgment.

Content validity was evaluated through expert review. An expert panel consisting of one board-certified emergency physician, two professors of paramedicine, and two paramedics with more than ten years of clinical experience assessed the items for relevance, clinical appropriateness, and clarity. Based on their feedback, items were refined to enable a multidimensional assessment of perceived competence in handling tasks, maintaining concentration, and managing work-related stress. Representative items included “I feel I have sufficient competence to handle my tasks,” “I can effectively relieve stress during work,” and “I am able to maintain concentration while working,” which were intended to capture both work efficiency and psychological stability.

After item refinement, the content validity index was calculated. All items met the predefined retention criterion and demonstrated an item-level content validity index of 0.81. A pilot test with 15 individuals who had characteristics similar to those of the study participants was conducted to evaluate item comprehensibility, response appropriateness, response patterns, and completion time. Feedback from the pilot test was incorporated into the final scale. In the present study sample, internal consistency reliability of the performance confidence scale was acceptable, with a Cronbach’s alpha of 0.875. Total scores ranged from 1 to 5, with 3 indicating a mid-level response.

#### 2.4.6. Statistical Analysis

To address the study objectives, data were analyzed using IBM SPSS Statistics version 23.0 with two-tailed tests and a significance threshold of *p* < 0.05. First, to quantify psychological changes associated with the resident doctors’ collective action, we compared pre- and post-scores within the same participants for outcomes that were measured at both time points, specifically job stress, job satisfaction, and performance confidence, using paired-samples *t* tests. For each outcome, we reported the pre- and post-means, the mean change, and the corresponding *p* value. Second, self-efficacy was analyzed as a pre–post outcome using paired-samples *t*-tests, consistent with other domains. Third, to evaluate whether psychological responses varied by career stage, we performed additional between-group comparisons of senior versus junior paramedics on post-period levels and on pre- to post-change scores for the repeated-measures outcomes, using independent-samples *t* tests when group comparisons were required. Finally, to examine the interrelationships among job stress, self-efficacy, job satisfaction, and performance confidence and to support the objective of verifying psychological responses as a correlated set of constructs, we calculated Pearson correlation coefficients among the scale scores. Prior to hypothesis testing, we computed scale scores by averaging item responses with reverse coding applied to negatively worded items, and we assessed internal consistency reliability for each multi-item scale using Cronbach’s alpha.

## 3. Results

### 3.1. General Characteristics

The results of the analysis of participants’ general characteristics are presented in Table 1. The mean age was 30.8 ± 12.81 years. In terms of sex, 102 participants (63.0%) were female and 59 (37.0%) were male. Regarding marital status, 113 (73.0%) were single, 42 (26.0%) were married, and 1 (0.6%) did not respond. By type of affiliated institution, 65 participants (40.3%) worked at regional emergency medical centers, whereas 96 (59.7%) worked at local emergency medical centers. For employment status, permanent employees accounted for the largest proportion (*n* = 103, 63.2%), followed by fixed-term contract employees (*n* = 53, 32.9%) and indefinite-contract employees (*n* = 6, 4.0%). With respect to the number of paramedics working in the same institution, the most common category was 10–20 (*n* = 56, 34.7%), followed by 20–30 (*n* = 47, 29.0%) and ≤5 (*n* = 16, 9.3%). Clinical experience was most frequently 1 to <3 years (*n* = 46, 29.2%), followed by 5 to <10 years (*n* = 43, 26.5%), ≥10 years (*n* = 36, 22.4%), <1 year (*n* = 20, 12.0%), and 3 to <5 years (*n* = 16, 8.3%) (Table 1).

### 3.2. Comparison of Job Stress Before and After the Resident Doctors’ Strike

The results of the comparison of job stress before and after resident doctors’ collective action are presented in Table 2. Respondents reported that they were more often assigned additional tasks before completing their current work, with the mean score increasing from 2.8 ± 0.9 before the strike to 3.6 ± 1.0 after the strike. Perceived workload also increased, with the score rising from 2.9 ± 1.0 pre-strike to 3.5 ± 1.0 post-strike. The perceived intensity of work requiring prolonged concentration increased from 3.0 ± 0.8 to 3.7 ± 0.9. In addition, the perception that sufficient rest was not provided increased from 3.1 ± 0.7 to 3.9 ± 0.9, and the item indicating the need to perform multiple tasks simultaneously rose from 3.3 ± 0.8 to 4.1 ± 0.9 (Table 2).

### 3.3. Pre–Post Comparison of Self-Efficacy Following Resident Doctors’ Strike

The results of the analysis of changes in self-efficacy among paramedics before and after the doctors’ strike are presented in Table 3. In the domain-level analysis, the mean score for the item stating that respondents could handle all assigned tasks was 4.1 ± 0.7 before the strike and 4.0 ± 0.9 after the strike. The item reflecting confidence in making plans and completing them showed a slight decrease from 4.3 ± 0.6 to 4.2 ± 0.8. The mean score for the item indicating that respondents did not avoid difficult tasks declined from 3.9 ± 0.9 to 3.6 ± 0.9, and the item stating that they did not feel anxious about their abilities while performing their duties also decreased from 4.2 ± 0.8 to 4.1 ± 0.9 (Table 3).

### 3.4. Comparison of Job Satisfaction Before and After Doctors’ Strike

The results of the comparison of job satisfaction among paramedics before and after resident doctors’ collective action are presented in Table 4. The mean score for the item “I feel a sense of accomplishment after finishing a day’s work” decreased from 3.9 ± 0.7 before the strike to 3.5 ± 0.9 after the strike. Satisfaction with the current workplace also declined from 3.6 ± 0.9 to 3.1 ± 1.0. Willingness to remain committed to the job showed a slight decrease from 3.8 ± 0.8 to 3.7 ± 0.9. In contrast, the item indicating turnover intention if better compensation and working conditions were available increased markedly from 2.7 ± 1.0 pre-strike to 3.9 ± 0.9 post-strike (Table 4).

### 3.5. Comparison of Performance Confidence Before and After Resident Doctors’ Strike

The results of the comparison of performance confidence among paramedics before and after the resident doctors’ collective action are shown in Table 5. The mean score for the item “I feel I have sufficient competence to perform my duties” was 4.4 ± 0.6 before the strike and 4.3 ± 0.8 after the strike. The item “I can concentrate on my work” showed a slight decrease from 4.1 ± 0.7 to 4.0 ± 0.9, and the item “I handle my work efficiently and skillfully” decreased from 4.3 ± 0.7 to 4.2 ± 0.8 (Table 5).

### 3.6. Comparison of Psychological Changes Between Senior and Junior Paramedics

**Figure 1:**
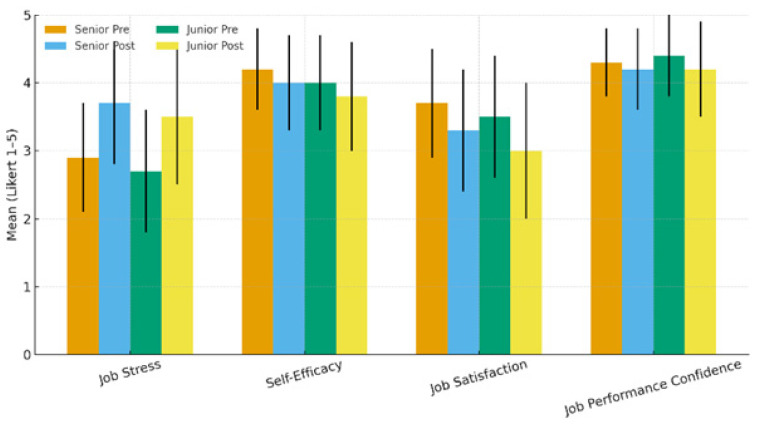
Changes in job stress, self-efficacy, job satisfaction, and job performance confidence before and after the resident doctors’ strike by paramedic seniority. Bars represent mean scores (5-point Likert scale) for senior (N = 81) and junior (N = 80) paramedics measured pre-strike and post-strike. Error bars indicate standard deviation (SD). Within-group pre–post comparisons were conducted separately for each seniority group.

To examine psychological changes by career stage, we compared pre–post mean scores for job stress, self-efficacy, job satisfaction, and performance confidence between the senior group (N = 81) and the junior group (N = 80). The results are summarized in Table 6. Job stress increased significantly in both groups. In the senior group, the mean score increased from 2.9 ± 0.8 pre-strike to 3.7 ± 0.9 post-strike (*p* = 0.001), and in the junior group, it increased from 2.7 ± 0.9 to 3.5 ± 1.0 (*p* = 0.003). Self-efficacy showed a small but significant decrease in both groups, declining from 4.2 ± 0.6 to 4.0 ± 0.7 in the senior group (*p* = 0.038) and from 4.0 ± 0.7 to 3.8 ± 0.8 in the junior group (*p* = 0.042). Job satisfaction also decreased significantly in both groups, from 3.7 ± 0.8 to 3.3 ± 0.9 in the senior group (*p* = 0.005) and from 3.5 ± 0.9 to 3.0 ± 1.0 in the junior group (*p* = 0.004). In contrast, performance confidence did not differ significantly before and after the strike in either group. The senior group changed from 4.3 ± 0.5 to 4.2 ± 0.6 (*p* = 0.214), and the junior group from 4.4 ± 0.6 to 4.2 ± 0.7 (*p* = 0.198), with neither change reaching statistical significance (Table 6) (Figure 1).

## 4. Discussion

Job stress increased significantly across nearly all items, with particularly prominent rises observed in increased workload, being assigned additional tasks before completing existing tasks, the need to maintain concentration for prolonged periods, performing multiple tasks simultaneously, and insufficient rest. These findings are consistent with prior reports describing heightened job stress among nurses during the COVID-19 pandemic and outbreak-related burnout driven by job stress in emergency settings [15,16].

In addition, nationwide data during COVID-19 indicate that paramedics and emergency medical technicians experienced significant psychological distress and increased willingness to leave their jobs after managing infectious patients, supporting the broader plausibility of outbreak-like stress amplification among EMS personnel [17].

However, the stress increase observed in the present study cannot be interpreted solely within the same category as general workload intensification during infectious disease crises.

In this context, job stress did not rise simply because there was more work, but because a substantial portion of clinical support tasks previously performed by residents was transferred to paramedics, thereby requiring advanced clinical knowledge, procedural competence, and more independent clinical judgment and accountability. Importantly, this qualitative burden maps onto the specific activities that expanded during the disruption and remained elevated afterward in many settings, particularly invasive bedside procedures and high-stakes workflow responsibilities that had commonly been performed by residents.

When such activities expand under time pressure, perceived legal and professional accountability increases, which plausibly amplifies job stress even beyond what would be expected from workload alone. This interpretation is supported by human-factors evidence showing that interruptions, multitasking, and poor sleep in emergency settings are associated with higher rates of clinical task errors, suggesting a safety-relevant pathway through which sustained multitasking and fatigue may affect care processes [18]. In addition to staff-centered outcomes, the role expansion and task redistribution observed in this study are likely to have influenced patient care processes in the ED.

Although patient outcomes were not directly measured, the combination of increased multitasking, sustained concentration demands, time pressure, and reduced rest suggests plausible pathways through which care delivery could have been affected [18].

This interpretation aligns with systematic evidence that emergency department crowding and throughput delays are associated with poorer patient outcomes and reduced ability of staff to adhere to guideline-recommended care, reinforcing the importance of monitoring ED quality indicators such as timeliness of key interventions, ED length of stay, and return visits when workforce disruptions occur [19]. Put differently, as tasks become more complex and require higher-level judgment, and as institutional and legal safeguards remain insufficient, stress is not merely accumulated but may be structurally amplified.

In Korea, legal and practical mismatches between the formally defined scope of practice and tasks performed in real settings have been documented in policy and legislative feasibility work on paramedic scope expansion, which provides a plausible institutional basis for accountability ambiguity during role expansion [20]. Internationally, task shifting and advanced-role implementation require explicit governance, including regulation, credentialing, and oversight to reduce practice variation and maintain quality and safety [20]. Global guidance likewise emphasizes formal task-shifting frameworks, structured training, supervision, and accountability mechanisms to support safe implementation during workforce shortages [21].

Professional policy statements also caution that task sharing and task shifting can introduce risks to quality and safety if scope, education, and governance are not clearly established [22]. Self-efficacy decreased in some domains after the strike.

This pattern is consistent with self-efficacy theory, which emphasizes that efficacy beliefs are sensitive to perceived mastery conditions, uncertainty, and contextual constraints affecting perceived capability [23]. In the present context, rapidly expanded responsibilities without formalized training and competency validation could plausibly suppress self-efficacy even when performance confidence in core skills remains stable. Job satisfaction decreased overall, and the post-strike increase in intent to leave suggests a sustained perception of imbalance between expanded responsibilities and institutional support.

Evidence from Korean EMS personnel during COVID-19 indicates that lack of organizational compensation for overtime work is associated with higher burnout, underscoring the relevance of reward and recovery structures when workload intensifies [24].

More broadly, a recent meta-analysis reports substantial prevalence of turnover intention among EMS personnel and highlights the role of mental health support, workload management, and professional development opportunities in improving job satisfaction and retention [25]. An interesting finding was that performance confidence did not show a statistically significant change.

This may reflect preservation of perceived clinical competence despite higher strain, which is consistent with evidence that performance confidence is shaped by training and accumulated practice exposure [26].

In addition, career-stage differences may reflect differential role expectations and leadership burden among seniors and adaptation-related stress among juniors, consistent with the broader expert-performance literature emphasizing the role of accumulated experience and practice demands in sustaining high-level performance under pressure [27,28]. These findings offer important implications for emergency system operations and health policy.

During workforce gaps, contingency plans should define which resident-dependent tasks may be shifted to paramedics, under what supervision and documentation requirements, and with what legal safeguards, and these plans should be accompanied by monitoring systems that track task redistribution and key ED performance indicators [19,20,21,22].

Competency-based credentialing systems for high-risk procedures should include standardized training, objective skills assessment, minimum supervised case numbers, and periodic revalidation, consistent with governance principles for task shifting and advanced roles [20,21,22]. This study has several limitations. First, the use of self-report surveys may have introduced subjective bias. Second, because pre-strike data were collected retrospectively based on recall rather than in real time, there are constraints on precise time-series interpretation. Third, because patient outcomes were not directly measured, the implications for patient safety and care processes remain inferential and should be tested in future studies using ED performance metrics [19]. Fourth, because the survey was administered during an ongoing period of operational adjustment, unmeasured concurrent stressors such as organizational conflict, policy uncertainty, or personal stress may have contributed to the observed psychological outcomes, limiting causal attribution solely to the strike.

## 5. Conclusions

Paramedics in the Republic of Korea play a pivotal role not only in the prehospital phase but also within emergency medical centers, serving as a key bridge in resuscitating critically ill patients and advancing the emergency medical system. In particular, following the resident doctors’ strike, paramedics in emergency medical centers worked to fill workforce gaps by meeting organizational demands and doing their utmost to minimize disruptions in care. As a result, job stress increased significantly, whereas self-efficacy and job satisfaction partially declined, and performance confidence tended to be maintained. These findings suggest that paramedics have faced an increased psychological burden under conditions of resident workforce shortages, underscoring the urgent need for legal and policy reforms, including role redefinition, the establishment of emotional support systems, and appropriate compensation frameworks. It is now a critical time for public support and government-led policy improvements to ensure that paramedics in emergency medical centers can remain fully committed to resuscitating emergency patients and dedicating themselves to their professional roles.

## Figures and Tables

**Table 1:** General characteristics of survey participants (N = 161).

Variables	N (%) or Mean ± SD
Age	30.8 ± 12.81
Gender	
Female	102 (63.0)
Male	59 (37.0)
Marriage	
Single	118 (73.0)
Married	42 (26.0)
No response	1 (1.0)
Emergency Medical Center Type Regional Emergency Medical Center Local Emergency Medical Center Employment Type Permanent Contract-based Indefinite-term contract Number of paramedics working in emergency medical center	65 (40.3) 96 (59.7)
<5	16 (9.3)
5–10	40 (24.8)
10–20	56 (34.7)
20–30	47 (29.0)
30<	2 (2.2)
Clinical experience	
>1 yr	20 (12.0)
1–3 yr	46 (29.2)
3–5 yr	16 (8.8)
5–10 yr	40 (25.5)
10 yr<	39 (23.5)
Paramedic seniority groups	
Senior paramedics group	81 (50.3)
Junior paramedics group	80 (49.7)

Variables are presented as N (%) or Mean and standard deviation.

**Table 2:** Comparison of job stress before and after the resident doctors’ strike (N = 161).

Variables	Pre-Strike (Mean ± SD)	Pre Level	Post-Strike (Mean ± SD)	Post Level	Δ (Post–Pre)	*p*-Value
Assigned additional tasks	2.8 ± 0.9	Normal	3.6 ± 1.0	High	+0.8 ↑	<0.001
Increased workload	2.9 ± 1.0	Normal	3.5 ± 1.0	High	+0.6 ↑	<0.001
Required prolonged concentration	3.0 ± 0.8	Normal	3.7 ± 0.9	High	+0.7 ↑	<0.001
Insufficient rest	3.1 ± 0.7	Normal	3.9 ± 0.9	High	+0.8 ↑	<0.001
Multitasking required	3.3 ± 0.8	Normal	4.1 ± 0.9	High	+0.8 ↑	<0.001

Values are presented as mean ± standard deviation (SD). Higher scores indicate greater job stress. Pre-strike and post-strike scores were compared using a paired-samples *t*-test (two-tailed). Scores were categorized as Low (1.00–2.49), Normal (2.50–3.49), and High (3.50–5.00) based on a 5-point Likert scale. Higher scores indicate greater job stress. Statistical significance was set at *p* < 0.05. Arrows indicate the direction of change from pre- to post-strike (Δ = Post–Pre): ↑ denotes an increase.

**Table 3:** Comparison of self-efficacy before and after the resident doctors’ strike (N = 161).

Variables	Pre-Strike (Mean ± SD)	Post-Strike (Mean ± SD)	*p*-Value
Able to complete all tasks	4.1 ± 0.7	4.0 ± 0.9	0.015
Confident in completing plans	4.3 ± 0.6	4.2 ± 0.8	0.019
Do not avoid difficult tasks	3.9 ± 0.9	3.6 ± 0.9	<0.001
No anxiety about abilities	4.2 ± 0.8	4.1 ± 0.9	<0.001

Values are presented as mean ± standard deviation (SD). Higher scores indicate greater self-efficacy (task performance confidence). Pre-strike and post-strike scores were compared using a paired-samples *t*-test (two-tailed). Statistical significance was set at *p* < 0.05.

**Table 4:** Comparison of job satisfaction before and after resident doctors’ strike (N = 161).

Variables	Pre-Strike (Mean ± SD)	Pre Level	Post-Strike (Mean ± SD)	Post Level	Δ (Post–Pre)	*p*-Value
Feel job rewarding	3.9 ± 0.7	High	3.5 ± 0.9	High	−0.4 ↓	<0.001
Satisfied with workplace	3.6 ± 0.9	High	3.1 ± 1.0	Normal	−0.5 ↓	<0.001
Willing to commit to job	3.8 ± 0.8	High	3.7 ± 0.9	High	−0.1 ↓	<0.001
Intent to leave for better conditions	2.7 ± 1.0	Normal	3.9 ± 0.9	High	+1.2 ↑	<0.001

Values are presented as mean ± standard deviation (SD). Higher scores indicate greater job satisfaction; for the item “Intent to leave for better conditions,” a higher score indicates stronger turnover intention. Pre-strike and post-strike scores were compared using a paired-samples *t*-test (two-tailed). Scores were categorized as Low (1.00–2.49), Normal (2.50–3.49), and High (3.50–5.00) based on a 5-point Likert scale. Statistical significance was set at *p* < 0.05. Arrows indicate the direction of change from pre- to post-strike (Δ = Post–Pre): ↑ denotes an increase, and ↓ denotes a decrease.

**Table 5:** Comparison of performance confidence before and after resident doctors’ strike (N = 161).

Item Description	Pre-Strike (Mean ± SD)	Pre Level	Post-Strike (Mean ± SD)	Post Level	Δ (Post–Pre)	*p*-Value
Have ability to perform tasks	4.4 ± 0.6	High	4.3 ± 0.8	High	−0.1 ↓	0.2481
Able to focus on work	4.1 ± 0.7	High	4.0 ± 0.9	High	−0.1 ↓	0.0521
Handle tasks efficiently	4.3 ± 0.7	High	4.2 ± 0.8	High	−0.1 ↓	0.4673

Values are presented as mean ± standard deviation (SD). Higher scores indicate greater performance confidence. Pre-strike and post-strike scores were compared using a paired-samples *t*-test (two-tailed). Scores were categorized as Low (1.00–2.49), Normal (2.50–3.49), and High (3.50–5.00) based on a 5-point Likert scale. Statistical significance was set at *p* < 0.05. Arrows indicate the direction of change from pre- to post-strike (Δ = Post–Pre): ↓ denotes a decrease.

**Table 6:** Comparison of psychological changes between senior and junior paramedics (N = 161).

Variables	Senior Group (N = 81)			Junior Group (N = 80)		
	Pre-Strike	Post-Strike	*p*-Value	Pre-Strike	Post-Strike	*p*-Value
Job Stress	2.9 ± 0.8	3.7 ± 0.9	0.001	2.7 ± 0.9	3.5 ± 1.0	0.003
Self-Efficacy	4.2 ± 0.6	4.0 ± 0.7	0.038	4.0 ± 0.7	3.8 ± 0.8	0.04
Job Satisfaction	3.7 ± 0.8	3.3 ± 0.9	0.005	3.5 ± 0.9	3.0 ± 1.0	0.004
Job Performance Confidence	4.3 ± 0.5	4.2 ± 0.6	0.214	4.4 ± 0.6	4.2 ± 0.7	0.198

Values are presented as mean ± standard deviation (SD). Higher scores indicate greater levels of each construct (job stress, self-efficacy, job satisfaction, and performance confidence). Within-group pre–post differences were tested separately for the senior and junior groups using paired-samples *t*-tests (two-tailed). Statistical significance was set at *p* < 0.05.

## Data Availability

The original data presented in this study are included in the article. The minimal dataset supporting the main findings is available from the corresponding author upon reasonable request. Further inquiries can be directed to the authors.

## Abbreviations

The following abbreviations are used in this manuscript:

ED	Emergency department
EMS	Emergency medical services
IRB	Institutional Review Board
CVI	Content Validity Index
I-CVI	Item-level Content Validity Index
SAS	Statistical Analysis System
SPSS	Statistical Package for the Social Sciences
SD	Standard deviation
COVID-19	Coronavirus disease 2019
N	Number of participants
